# partition: A fast and flexible framework for data reduction in R

**DOI:** 10.21105/joss.01991

**Published:** 2020-03-18

**Authors:** Malcolm Barrett, Joshua Millstein

**Affiliations:** 1 Department of Preventive Medicine, University of Southern California

## Summary

Data are increasingly feature-rich, including many variables for each observation; in modern genomics, for example, high-resolution genetic data captures much more information than it did just a decade ago. While improved measurements contribute immensely to science, they also increase computational burden and complicate interpretability (Karczewski & Snyder, 2018). Data reduction techniques, such as principal component analysis (PCA) and K-Means clustering, are vital tools used to address these issues, particularly for noise and redundancy. However, these techniques may lead to problems in scalability, information loss, and interpretability (Malod-Dognin, Petschnigg, & Pržulj, 2018). The Partition framework is an approach to data reduction that is flexible, scalable, and interpretable, developed to address information loss while maintaining speed (Millstein et al., 2020). As opposed to other data reduction strategies, Partition only reduces data if the data reduction retains a specified amount of information. This framework is also agnostic to how partitions form; users can easily use other tools such as PCA and t-Distributed Stochastic Neighbor Embedding (t-SNE) to create a partition or summarize data within a partition subset and thus reduce data while constraining information loss.

The package partition is a fast and flexible data reduction tool that implements the Partition framework for the statistical programming language R, available for download on CRAN (R Core Team, 2019). partition enforces a minimum level of information, specified by the user, that reduced features must capture. Each feature begins as an independent cluster. Then, a bottom-up (agglomerative) approach grows clusters as much as possible, subject to the information loss constraint. partition then summarizes each feature subset into a single new feature. The reduced features are highly interpretable because original features map to one and only one feature in the reduced data set. The partition software is flexible and customizable in the way features are agglomerated, information is measured, and data are reduced. Additionally, we have thoroughly benchmarked and profiled partition, with critical components written in C++ to improve performance.

partition uses an approach we call Direct-Measure-Reduce to modularize the Partition framework, facilitating the speed and flexibility of the data reduction process. These three components (directors, metrics, and reducers), collectively called partitioners, tell the partition algorithm (1) how to partition data, (2) how to measure information loss when reducing data, and (3) how to summarize partition subsets into reduced features, respectively. partition has several pre-specified partitioners for data reduction, but this approach is also quite flexible.

The default partitioner uses a correlation-based distance matrix to find the pair of features with the smallest distance between them; intraclass correlation (ICC) to measure information explained by the reduced variable; and scaled row means to reduce variables with a sufficient minimum ICC. In simulations, this ICC-based partitioner outperformed K-means and other approaches both in terms of speed and number of discoveries (Millstein et al., 2020). The ICC approach is fast and reliable, but partition also implements different strategies for directing, measuring, and reducing. For instance, the user can use principal component analysis to reduce variables instead of scaled row means. As the framework is agnostic to how partitions are being directed, measured, or reduced, custom partitioners are easy to implement.

Many tools exist in R for data reduction, including:

- In the base R stats package, princomp() and prcomp() for PCA, kmeans() for K-Means Clustering, and hclust() for Hierarchical Clustering, among others (R Core Team, 2019)
- cluster, an R package (recommended by CRAN) for grouping data using Hierarchical Clustering and other strategies (Maechler, Rousseeuw, Struyf, Hubert, & Hornik, 2019)
- Rtsne and tsne, R packages for t-SNE analysis (Donaldson, 2016; Krijthe, 2015)
- uwot, an R package for Uniform Manifold Approximation and Projection (UMAP) dimensionality reduction (Melville, 2019)

partition differs from these tools mainly in that it constrains information loss; the way features map to reduced data is more interpretable than many other data reduction approaches. Notably, because in the Partition framework the components of Direct-Measure-Reduce are easily interchangeable, users may apply tools from base R or other R packages at any of the three stages of the partition.

partition offers a fast and flexible tool that addresses the need for interpretability and information retention in reduced variables. To our knowledge, it is the first package of its kind. Feature-rich data, such as high-resolution genetic data, can be quickly reduced without sacrificing information. Additionally, as each feature of the data maps to a single reduced feature, it is easier to make inferences from the reduced data set. The flexibility of partition makes it adaptable to the needs of many data reduction strategies.
